# Round the Hospitals

**Published:** 1918-03-30

**Authors:** 


### ROUND THE HOSPITALS.

The great attack is in progress, and all minds are filled with an overpowering sense of the soldiers' ordeal. That they are not alone in their great need is the only source of comfort which the nation can find. The mighty chain of succour which stretches from close behind the lines right across to the peaceful villages in this country is the response which the nation makes to the mute appeal from the battlefield. We cannot forget that very many of the doctors and nurses and their assistants are directly exposed to the perils of shot and shell. The bombardment, so we are told, extends far beyond the fighting lines to a distance never before reached, owing to the use by the Germans of special high-power guns. What this means through the sudden evacuation of casualty-

☛ Previous articles appeared Dec. 8, 15, 22, Jan. 5, 12, 26, Feb. 2, and March 2, 9 and 23, p. 533.

**ROUND THE HOSPITALS—(*continued*).**

clearing stations and the transport of the wounded under fire some of our readers doubtless know from bitter experience. It is under such conditions that valour is revealed as no special possession of the constitutionally brave, but as the normal quality of the men and women of our race. Many who have themselves been in action testify to the fact that, compared with the fierce ardour of attack, the slow waiting time when shells are falling nearer and nearer every minute is an infinitely greater test of courage. And all this is going on while we are eating and drinking, sleeping and (actually) enjoying the sunshine, even taking an interest in the little things about us.

---

THE fathomless power for comfort which hangs over Passion-tide has never been more keenly experienced than this year, when the Passion of our Lord transfigures and illuminates the agonies of the battlefield. In every institution there are some who have near relatives at the Front. In nearly all there are nurses who have gone forth from the walls to take part in the great struggle against evil now in progress. Is there not in every one among us an intense desire, amounting at times to pain, to be helping our comrades in danger in the only possible way, by prayer, in silence? In some hospitals where the chapel is a real centre of religious life the nurses gather by twos and threes to offer silent intercessions for those in extremity of danger. We could wish that wherever nurses are working occasions of meeting together might be arranged where, without formal service, without even verbal utterance, they might kneel and remember those who are travailing in the crisis of their fate. The fellowship of silence stretches out into a region where no distinction of creed makes itself felt. High Church and Low Church, Catholic and Nonconformist, even some who have no very firm hold on any definite creed, may kneel together in silence without attempting to offer a list of petitions, and rise strangely strengthened and comforted. Who has not had evidence of more than one kind that those "out there" passionately desire our prayers? Let us give them what we can, a few moments of utter concentration on their needs at intervals during the day, a wireless message of faith in their valour, a sustaining consciousness of the Divine life encompassing them and us, before which the shades of death and evil pale and disappear.

---

A STRONG representative Committee has now been formed to make arrangements for the service in memory of those nurses who have fallen in the war. The Chairman is the Honourable Sir Arthur Stanley, G.B.E., C.B., M.P., and the other members of the Committee are Miss Becher, R.R.C., Matron-in-Chief Q.A.I.M.N.S.; Miss M. E. Goodall-Copestake, Superintendent Sister Q.A.R.N.N.S.; Miss Sidney Browne, R.R.C., Matron-in-Chief T.F.N.S. The Colonies are represented by Miss Wilson (Australia), Miss Macdonald (Canada), Lady Gladstone and Miss Jackson (South Africa), and Miss Thurstan (New Zealand). Miss Swift represents the British Red Cross Society and the Order of St. John of Jerusalem, while Lady Ampthill and Lady Perrott represent the Voluntary Aid Detachments. The Hon. Secretary and Organiser is the Rev. A. Lombardini, Church of St. Elizabeth, Kensington, W. 8., to whom all inquiries should be addressed.

---

IT has been definitely settled that the service will take place at St. Paul's Cathedral on Wednesday, April 10, at 2.30 P.M. The musical direction of the service will be in the hands of Major J. Mackenzie Rogan, M.V.O., with the band of the Coldstream Guards in attendance. Nurses in uniform and relatives of those in the Roll of Honour will have spaces reserved for them in the cathedral. It is very important to bear in mind that all nurses who wish to be present should apply to Mr. Lombardini for a ticket or they will find themselves, even though in uniform, among the crowds of the general public.

---

IT is not too early for heads of institutions to take measures for the collection of details about the war work of nurses who have been trained under them or have worked in their wards. Every hospital and infirmary should have its own Roll of Honour, which will come in the end to be handed down as one of the historic relics of the establishment. For the compilation of the section in this book relating to nurses who have fallen in the war or distinguished themselves by acts of conspicuous bravery, the matron must be responsible. To keep a full record demands often considerable effort in the collection of evidence, because when once the link which binds the nurse to the institution is broken there may be no one at hand to furnish particulars. But it is work which will amply repay the effort needed to ensure accuracy, and such records are greatly improved in value and interest if photographs are appended.

---

IT will be remembered that some short time back we collected and published information from nearly all the leading hospitals in the kingdom relating to the nurses they had sent forth to military or naval duties. The results were extremely stirring. But a great difference was observable as between one hospital and another in the manner in which these records had been kept. In some hospitals the career not merely of present but of long-past nurses doing war work had been hunted out with loving care, and no trouble had been spared to keep the memory green of the old friends. In such cases it was easy to realise that a corresponding meed of loyalty and recollection bound the nurses, after many years of nursing vicissitudes, to their *Alma Mater*. It is hard to estimate whether those who give or those who receive this gift of sympathetic remembrance are the more blessed. But there can be no two opinions about the romance which it confers on grey walls and days of drudgery.

**ROUND THE HOSPITALS—(*continued*).**

THE discussion in the *Times* about the well-being of nurses in France begins to touch upon the ludicrous, particularly in the matter of catering. Every sensible person knows that the officers' mess varies inevitably from quite good to unspeakably bad according to the locality, time of year, and so on. The mess president, often a junior lieutenant, is liable to incur a good deal of whole-hearted abuse even when he is quite clever at his job. Sisters and nurses put up philosophically with vicissitudes in this direction, much as their comrades-in-arms are compelled to do, and cannot fail to be a trifle amused when they hear that the deficiencies in their menu are due, on the one hand, to the high-handed appointment by their matron of a housekeeping sister, or, on the other, to the selection of a " mere V.A.D. " for this invidious office. It is precisely for housekeeping and similar duties that the V.A.D. workers are so invaluable, and Miss McCarthy has been recently testifying to their fine success in smoothing the lot of the nurses in this respect and relieving them of domestic labours both when on duty and in their intervals of rest. It seems incredible that people should still be found to suppose that sisters and nurses have leisure as a rule to do their own catering under the stress of war nursing, and that anyone should be sufficiently irresponsible to cast a stigma on the whole Nursing Service commissariat on the strength of isolated instances of incapacity on the part of a caterer new to the business. Whatever may be thought of the expediency of having a trained nurse for a housekeeper in institutions at home, such considerations cannot hold force for a moment abroad and in war. It is not the possession of high certificates which will enable their talented owner to discriminate between tough goat and tender mutton, or impart that genius for tracking down supplies which belongs to the old campaigner.

THE meeting on behalf of the Nation's Fund for Nurses, held at Plymouth on the 21st inst., was an unqualified success. The audience was thoroughly representative, including, as it did, Miss Margaret Priestman, R.R.C., Matron of the 4th Southern General Hospital, Ford House; Miss Holliday, Matron of the Infirmary, Greenbank; Miss Bridge, Matron of the Royal Albert Hospital; Miss Dickson, Matron of the South Devon and East Cornwall Hospital; Miss Terry, Lady Superintendent of the Three Towns Nursing Association; Miss Kearsey, Matron of the Pearn Convalescent Home; and a very large number of sisters and nurses from the military, territorial, and civil hospitals in the Three Towns. The success of th. gathering was entirely due to the organising ability of Miss Tait McKay, R.R.C., Matron of the 4th Southern General Hospital, Salisbury Road, who will, we trust, be encouraged by the cordial response of her colleagues and by the enthusiasm of the meeting to form very shortly a Centre of the College of Nursing at Plymouth. There is hardly any place in England where more nurses are congregated or where a Centre of nursing interests is more needed.

THE decision to establish, as part of the Nation's Fund for Nurses, a Tribute Fund available for the relief of all nurses, irrespective of their connection with the College, will be welcomed by many of its warmest supporters. There are always two classes of givers. The one prefers to support endowments and building schemes such as confer a permanent character on their gifts. The other prefers to seek out cases of individual distress and enjoy the knowledge that someone is definitely the happier for their benevolence. The Nation's Fund for Nurses now meets both classes. On the one hand it points to the need for the permanent endowment of a College of Nursing if nurses are to be placed on the same footing of strength as members of other professions, in the matter of combining for professional purposes. On the other hand it invites and can dispense with wisdom and liberality the offerings of all who want to come between nurses and the vicissitudes of fortune. That any nurses, who have spent themselves in saving life, should, in the end, come short of the barest necessaries is intolerable. The Tribute Fund should render it impossible. The Council of the College is on very strong ground when it refuses to limit the application of the Fund solely to those who have had the prudence and public spirit to join the College.

WE have received a letter, the substance of which, after full consideration, we feel it our duty to publish. It gives an account of the alleged state of affairs in the Essex County Hospital, Colchester, which, if it exists, must be ended without a moment's avoidable delay. We can only conclude that neither the committee nor the medical staff can realise the possibility of such a condition of affairs existing in any institution for which they are in whole or in part responsible. Our correspondent should be in a position to know all the facts, and has shown public spirit in sending them to the Editor. Up to the commencement of the war the hospital in question had a good name through its training-school. Its matron was called up for military service, and until the spring it was in charge of a sister whose indifferent health interfered with the discharge of her duties. Subsequently an untrained Commandant was in charge, and conditions became " deplorable." Before the end of six months a matron worthy of the position took office. She proved to be an excellent woman, but did not possess, it is stated, the assistance of the committee and the honorary and resident staff in every possible way to bring the hospital up to the standard it once held, that of " an excellent training-school." Instead, things were made as difficult as possible for the matron, and in the course of three months five sisters left. This was followed by the resignation of the matron and three more of the sisters. The resident staff is said to have consisted of two coloured lady doctors, whose treatment of the sisters is declared to have been reprehensible, and their reports to the committee and honorary staff open to challenge. There has been an absence of decorum, and of the exhibition of a reverent regard for life in the operation theatre, it is declared, and

**ROUND THE HOSPITALS—*(continued).***

the non-enforcement of strict asepsis has been followed by septic trouble. There is stated to have been a serious shortage of ward linen, and generally, if the condition of affairs throughout the Essex County Hospital, Colchester, is as stated, it calls for an independent investigation by the governors, under the chairmanship of a gentleman of experience, with a knowledge of public business and legal inquiries, and an earnest determination to secure the restoration of the high standard which formerly prevailed.

THE public inquiry into the administration of the Beckett Hospital, Barnsley, has now begun, its object being to investigate "with regard to the administration of the hospital generally, and in particular to the cause of unrest (if any) amongst the nursing staff during the past eighteen months." The inquiry has been instituted owing to the outstanding fact that within twelve months eight sisters and no fewer than thirty-two nurses have left the hospital. This is really remarkable when it is remembered that the hospital has an average of ninety-one occupied beds, and is staffed by five sisters and eighteen nurses and probationers. Mr. Pawsey, honorary secretary of the hospital, on behalf of the House Committee, at whose instance the inquiry is being held, opened the proceedings by asserting that the committee, "with possibly one dissentient, had no cause to consider that the management of the hospital was not perfectly efficient and satisfactory," and believed "the hospital had been conducted as well as possible in these difficult times." Everyone acquainted with hospital abuses knows the traditional attitude of a committee perfectly satisfied with things as they are. Mr. Feasby, a member of the House Committee and chairman of the Saturday and Sunday movement, revealed himself as the "possible dissentient," and had some strong things to say about the action of the committee with regard to the provision of increased sleeping accommodation for the staff. If it be true, as the chairman of the House Committee stated, that before the present matron, Miss Burkhill, came "there was a lot of unruly girls on the staff," we think the committee would be wise to extend their inquiry into the rate of pay, hours on duty, facilities for recreation, housing, and the general well-being of the probationers, so that light may be thrown on the reasons why the Beckett Hospital has been able to attract only the lower stratum of candidates. The inquiry is still proceeding.

THE newest resource for the institution housekeeper worried by the "fat" shortage is coco-butter, which is on sale in some parts of the country at a reasonable price. The flavour is strong, but it may be clarified in the usual way, and is then a very good medium for frying. It becomes extremely hard after it has been clarified, and for pastry- or cake-making needs grating, but if this extra trouble is not objected to coco-butter is a great boon. Coco-nut and cashew-nut butters, made of the crushed nuts mixed with oil, are an acceptable substitute for butter on bread. They can be obtained at most vegetarian depôts and by many epicures are said to be preferred to what we are now driven to call "cow butter."

IT is our invariable practice to pay for all items of news which we may publish. The minimum payment is 5s. Paragraphs of about twenty lines in length—that is to say, of 150 words or less—are preferred. Contributions should be addressed to The Editor, THE HOSPITAL, 28 and 29 Southampton Street, Strand, London, W.C. 2, and, to be in time for the current week's issue, should reach him by the first post on Mondays.

